# Evaluation of the Impact of Smoking on Orthodontic Treatment

**DOI:** 10.7759/cureus.73431

**Published:** 2024-11-11

**Authors:** Simi S, Reshma Mohan, Deepak Chandrasekaran, Deenadayalan Purushothaman, Akshay Tandon, Nidhi Angrish, Praveen Katepogu

**Affiliations:** 1 Orthodontics and Dentofacial Orthopaedics, Sri Ramaswamy Memorial (SRM) Kattankulathur Dental College and Hospital, SRM Institute of Science and Technology (SRMIST), Chengalpattu, IND

**Keywords:** orthodontic treatment, periodontal health, smoking, smoking cessation, tooth movement

## Abstract

Smoking is a prevalent habit known for its detrimental effects on oral health and it affects not only oral health but also various organ systems. The influence of smoking on periodontal health, including gingival inflammation, bone loss and delayed wound healing, poses significant challenges during orthodontic therapy. Orthodontic treatment depends on healthy bone and periodontal remodelling. Smoking reduces blood flow and oxygen to these areas, which can slow tooth movement and complicate treatment. It also increases plaque buildup, leading to potential periodontal diseases and enamel damage. Even after treatment, smoking can affect how well your teeth stay in their new position, increasing the risk of relapse. Quitting smoking can help improve your results and keep your smile healthier in the long run. Therefore, the literature concluded that orthodontists should consider smoking cessation interventions as an integral part of patient management to optimize treatment success and long-term oral health. This review explores the specific implications of smoking on orthodontic treatment outcomes.

## Introduction and background

Tobacco use is associated with an increased risk of periodontal diseases, loss of teeth, and diminished success in orthodontic treatments, which may result in loss of tooth movement. Most individuals begin smoking at a very young age, and due to many unsuccessful attempts at quitting, prevention is crucial. Nicotine is just one of the 7,000 harmful components found in tobacco. Numerous studies have investigated the effects of nicotine on bone remodelling using various assessment methods [1]. 

Orthodontic treatment can lead to changes in alveolar bone thickness, potentially causing defects or perforations depending on the movement pattern. This process involves biological reactions that remodel the bone, but the resulting low mineralization can weaken bone structure, increasing the risk of complications like intrusion and tipping. Factors such as age, sex, genetic predisposition, and lifestyle choices, including smoking, can affect bone mineral density (BMD) [2].

Orthodontics aims to enhance the stomatognathic system's function, aesthetics, periodontal health, and treatment stability, with aesthetic improvement being a key motivator for patients. Patients seek aesthetic brackets made from translucent plastic or ceramic for their colour stability. While ceramic brackets typically resist staining, plastic ones can discolour due to exogenous factors like food and beverages and endogenous factors like UV radiation. Smoking introduces hazardous materials, such as tar and carbon monoxide, into the oral cavity, potentially staining teeth and resin composites. The global prevalence of smoking, including in Brazil, raises concerns about the potential discolouration of aesthetic brackets due to cigarette smoke and heat [3].

Environmental factors, including smoking, which affects around one billion people worldwide, can influence the bond between the resin and the enamel. Tobacco smoke, a dynamic aerosol containing up to 5600 chemicals, can lead to surface roughness, hardness changes, and discolouration of enamel. High temperatures from smoking may also alter adhesive resin properties, compromising the chemical and mechanical bond between composite resin and enamel [4]. Tobacco smoke can impact microorganisms' adhesion and biofilm formation on orthodontic materials [5]. Young adults and teenagers frequently engage in smoking, which poses a significant concern for orthodontists due to the known severely compromised periodontal health of smokers compared to non-smokers [6].

The main aim was to explore the difficulties orthodontic patients encounter when they smoke, describing the biological effects of smoking on their care and providing coping mechanisms. In order to improve treatment outcomes, lower problems, and improve orthodontic patients' long-term periodontal health, it highlights how crucial it is to address smoking habits.

## Review

Methodology

Keywords like "orthodontic," "smoking," "effect of smoking," and "color stability of orthodontic appliances" were utilized to search platforms such as Google Scholar, Science Direct, PubMed and Sage Journal for pertinent clinical research published in English up to 2024. We performed a comprehensive review of published research. After a comprehensive review of the reference list, we identified pertinent papers for analysis and examined their references for more related research.

Discussion

Influence of Smoking on Periodontal Health

Orthodontic treatment may improve, harm, or preserve the periodontal condition. Patients having orthodontic therapy may have an increased risk of periodontal problems. Thus, before beginning orthodontic treatment, a comprehensive periodontal evaluation is necessary [7].

It is thought that smoking's effects come from nicotine's ability to narrow blood vessels and activate platelets, carbon monoxide's ability to cause low oxygen levels, and hydrogen cyanide's ability to stop cells from using oxygen for energy [8]. Jutberger et al. conducted a study to investigate the relationship between smoking and BMD, employing radiography to examine new fractures and prevalent vertebral fractures in older adults. The research determined that smoking among elderly men correlates with diminished BMD and a heightened incidence of vertebral and hip fractures [9].

Smoking is a major lifestyle characteristic that increases the risk of bone loss and fractures without regard to other variables such as age, weight, sex, or menopause [10].

Rosa et al. conducted a study comparing smokers and non-smokers (mean age: 20.5 years) to analyze the height and density of the alveolar bone. The study found that smoking negatively impacts clinical periodontal indicators and poses a potential cause of bone loss, even in young individuals with minimal tobacco use. Informing young smokers about these risks is crucial for their periodontal health awareness [11]. Cigarette smoking is associated with decreased BMD and an increased risk of fracture. Numerous clinical and observational investigations have identified various physiological mechanisms behind bone loss in smokers. These mechanisms include alterations in the metabolism of calciotropic hormone and intestinal calcium absorption, disruptions in the production and metabolism of sex hormones, changes in the metabolism of adrenal cortical hormone, and effects on the OPG system, which includes receptor activator of nuclear factor kappa-B ligand, osteoproteopterin, and nuclear factor kappa-B receptor activator. Additionally, smoking directly affects bone cells [12].

Smoking reduces bone mass and is associated with the onset of osteoporosis, especially in older individuals [13]. Rhee et al. carried out a cross-sectional study, which revealed that risk factors for middle-aged Korean men's declining BMD include greater age, lower BMI, lower blood IGF-I levels, and a history of current smoking [14].

The study by Sahtout et al. showed that tobacco harms cortical bone density, reducing it by 23.5% in smokers and 17.2% in nonsmokers [2]. Nicotine's effect increases the production of osteolytic mediators such as interleukins 1 and 8, RANK, and RANKL, promoting osteoclast activity, while downregulating osteoprotegerin, which impacts osteoblasts [15-17]. Research by Timock et al. shows that CBCT images accurately measure the height and thickness of the alveolar ridge when compared to direct measurements. Agreement between the two methods was higher for the measurements of buccal bone height than buccal bone thickness, as demonstrated by concordance correlation coefficients of 0.98 and 0.86, respectively [18].

Colour Stability

Discolouration can significantly affect the cosmetic value of aligners, making colour stability crucial. Researchers have thoroughly studied the stain resistance of thermoformed aligners in the past, attributing colour stability to both the production technique and material qualities [19].

Surface changes to aesthetic orthodontic wires can affect their visual appearance, potentially having a negative aesthetic impact and limiting acceptance among patients and professionals. These adjustments can impact both the appearance of the wires and their effectiveness in tooth movement. Cigarette smoke has documented its impact on aesthetic biomaterials, including orthodontic brackets, with approximately one billion smokers worldwide and tobacco use prevalent among teenagers. The in vitro study by Copello et al. revealed that exposure to cigarette smoke can alter the mechanical and optical properties of aesthetic orthodontic wires due to the colour-changing effect of smoke and the increased surface roughness of these wires [20]. The staining or discolouration of aesthetic orthodontic ligatures poses a clinical challenge since ceramic brackets and accessories may display varying degrees of colour stability compared to the ligatures. This discrepancy can lead to colour changes in the ligatures, affecting their appearance and possibly requiring more regular patient visits for the replacement of ligatures, which lengthens the clinical visit. When these materials interact in relation to the oral environment, they are impacted by various elements, such as nutrition, mouth rinses, toothpaste, brushing, temperature and pH changes, salivary components, biofilm accumulation, cosmetics, and smoking. These factors can cause both physical and chemical wear and tear on orthodontic appliances. An in vitro study by Miranda et al. revealed that orthodontic elastic ligatures exposed to cigarette smoke experienced a decrease in mechanical strength and changes in colour stability [21]. Factors such as exposure to staining agents, total exposure duration, and the brand of the product are influential in determining the colour stability of clear elastic ligatures [22].

Smoking and Periodontal Health in Orthodontic Treatment

Water pipe smoking has a similar impact on periodontal health as cigarette smoking does. This association appears to be independent of the subgingival microflora. Suzan Bakur Natto et al. conducted a comparative study between cigarettes, water pipe smokers, and non-smokers, and found that tobacco smoking adversely affects periodontal tissues, leading to loss of periodontal bone, pockets of periodontal tissue, and attachment loss. Researchers have observed a correlation between tobacco smoking and a decrease in periodontal bone height, and the effects of water pipe smoking are similar to those of cigarette smoking [23,24]. Smoking impairs inflammatory and immune responses to periodontal pathogens and exerts both systemic and local effects. Smokers have an increased prevalence and severity of periodontal disease. People who smoke are more likely to get acute necrotizing ulcerative gingivitis. They are also more likely to get periodontal disease, which causes pockets to form, attachment loss, and bone loss in the alveolar region. Cigar, pipe, waterpipe, and cannabis smoking have similar adverse effects on periodontal health as cigarette smoking. Passive smoking is also an independent periodontal disease risk factor. Smokeless tobacco is associated with localized periodontal disease. Smokers respond less favourably to both non-surgical and surgical treatments and have higher failure rates and complications following dental implantation. Smoking cessation may halt the disease progression and improve the outcome of periodontal treatment [25]. Enzyme-linked immunosorbent assay (ELISA) evaluates the negative effects of nicotine and tobacco exposure, as measured by blood cotinine, to diagnose reduced BMD. This approach is readily available, economical, and effective for diagnosing osteopenia and osteoporosis. Procrastination in these steps might result in significant difficulties since bones may become brittle and susceptible to fractures. Timely identification of serum cotinine can enable extensive community surveys to ascertain the magnitude of the problem. Research demonstrates that tobacco consumption adversely affects BMD, with smokeless tobacco exhibiting especially detrimental consequences [26].

Nicotine, the primary addictive element in tobacco, exerts numerous detrimental effects on oral tissues. It induces vasoconstriction, reducing blood flow and oxygen delivery to the gums and other oral tissues, hindering the healing process, and elevating the risk of periodontal disease. Furthermore, nicotine influences fibroblasts and keratinocytes in the gums, leading to diminished tissue regeneration and increased vulnerability to infections [27,28].

Effect of Smoking on Orthodontic Miniscrews

Although smoking constitutes a risk factor for implant failure, it is not an unequivocal contraindication. Clinicians must acquire a comprehensive smoking history, including length, intensity, and current status, prior to implant treatment. Dentists ought to advocate for smokers to quit, as smoking exacerbates difficulties and diminishes success rates. Clinicians must evaluate the hazards before proceeding, and informed permission is necessary prior to treatment [29]. According to research by Bauss and Bayat, 18.2% of orthodontic miniscrews (n = 20) failed overall. There was no significant difference between light and non-smokers, but heavy smokers had a significantly greater failure rate than light (p = 0.005) and non-smokers (p < 0.001) users. Within the first four months of insertion, the miniscrews in the heavy smoker group showed a significantly greater failure rate than those in the light smoker (p = 0.008) and non-smokers (p < 0.001) groups. These results suggest that heavy smoking negatively impacts the success rates of orthodontic miniscrews [30].

Risk Factor for Tobacco Susceptibility in Orthodontic Patients

Most smokers begin the habit during adolescence. Given that most attempts to quit are unsuccessful, prevention seems essential. Prevention is crucial as most smokers begin during their teenage years and attempts to quit often fail. An exploratory study by Jashinsky et al. included a cross-sectional sample of orthodontic patients and identified risk factors for smoking and vulnerability to starting to use tobacco products. Children in orthodontic groups appear to be more susceptible to smoking because of peer, familial, and environmental variables. By addressing these variables, dental professionals may be better able to recognize at-risk youth and offer helpful anti-smoking advice. Further prospective and experimental research is necessary to verify the potential role of dental clinicians in preventing youth smoking [31]. Chemicals in tobacco smoke impair the immune response in the oral cavity, making it more difficult to combat infections and recover from dental procedures [32].

Most participants, aged 18-25, were smokers and experienced prolonged treatment durations [1]. Due to the extended orthodontic preoperative treatment period in orthognathic surgery, there is a valuable opportunity to encourage and assist patients in quitting smoking, thereby reducing the risk of postoperative infections [33].

## Conclusions

Smoking greatly delays the healing process, aggravates periodontal disease, and raises the possibility of problems during orthodontic treatment, including bone loss and root resorption. Compared to non-smokers, these consequences result in longer treatment durations and higher relapse rates. Because smoking alters oral conditions, smokers are more likely to suffer problems with orthodontic appliances, such as bracket detachment, and decreased treatment effectiveness. Smoking's adverse effects on bone and periodontal health impair the long-term stability of orthodontic treatment outcomes. Smokers have higher relapse rates and require more intensive retention protocols.

Nicotine and other tobacco toxins impair blood flow, reduce oxygen supply, and weaken immune defenses, heightening infection risks and slowing healing. This leads to greater susceptibility to periodontal disease, bone loss, and complications in orthodontic procedures, such as higher miniscrew failure rates and discolouration of aesthetic appliances. Waterpipe and passive smoking have similar effects, while smokeless tobacco causes localized issues. Smoking cessation is crucial, especially for adolescents, to improve treatment success and long-term periodontal health.
